# Natural Genotypic Variation Underpins Root System Response to Drought Stress in Bambara Groundnut [*Vigna subterranea* (L.) Verdc.]

**DOI:** 10.3389/fpls.2022.760879

**Published:** 2022-03-28

**Authors:** Kumbirai Ivyne Mateva, Hui Hui Chai, Sean Mayes, Festo Massawe

**Affiliations:** ^1^Future Food Beacon, School of Biosciences, University of Nottingham Malaysia, Semenyih, Malaysia; ^2^Crops for the Future, National Institute of Agricultural Botany, Cambridge, United Kingdom; ^3^School of Biosciences, University of Nottingham, Leicester, United Kingdom

**Keywords:** drought stress, root length density (RLD), stomatal conductance (g_s_), bambara groundnut [*Vigna subterranea* (L.) Verdc.], root phenotyping

## Abstract

Bambara groundnut [*Vigna subterranea* (L.) Verdc.] is grown in rainfed production systems and suffers from periodic drought stress (DS), leading to yield reductions. Natural genotypic variation for root traits is essential for adaptation to water deficit conditions. However, root traits have not been fully utilised as selection criteria to improve DS in bambara groundnut. The present study explored the natural genotypic variation found in single genotypes of bambara groundnut derived from landraces to identify adaptive differences in tap root length (TRL) and root length density (RLD) in response to DS. A diverse core collection of eight bambara groundnut genotypes from various locations (namely, Gresik, LunT, IITA-686, DodR, S19-3, Tiga nicuru, and Ankpa-4, DipC1), were grown for two seasons (2018 and 2019) in polyvinyl chloride (PVC) columns with well-watered (WW) and 30-day DS treatments. Plant samples were collected at 55 days after emergence (DAE) (30 days of DS) and at 105 DAE (30 days of DS plus 50 days of recovery). Under DS, differential TRL among genotypes at 55 DAE was observed, with DodR recording the longest among genotypes with an increase (1% in 2018) in TRL under DS compared to WW, whereas LunT and IITA-686 showed significant (*p* < 0.001) decrease in TRL (27 and 25%, respectively, in 2018). Average RLD was observed to have the highest reduction under DS in the 90–110 cm layer (42 and 58%, respectively, in 2018 and 2019). Rainy habitat LunT had limited roots in 2018 and recorded the least (0.06 ± 0.013 cm^–3^) RLD in 2019. However, dry-habitat DodR showed an increase in the RLD (60–90 cm) under DS compared to WW, while dry-habitat S19-3 densely occupied all depths with RLD of 0.16 ± 0.05 and 0.18 ± 0.01 cm cm^–3^ in the deepest layer in both seasons, respectively. Reduced RLD under DS showed recovery when the plants were re-watered. These plants were additionally observed to have RLD that surpasses the density in WW at all soil depths at 105 DAE. Also, recovery was shown in Tiga nicuru and DodR (0–30 cm) and IITA-686 (90–110 cm) in 2019. Average RLD under DS treatment was associated with substantial grain yield advantage (*R*^2^ = 0.27 and *R*^2^ = 0.49, respectively) in 2018 and 2019. An increase in TRL allowed DodR to quickly explore water at a deeper soil depth in response to gradually declining soil water availability. High RLD in genotypes such as DodR, DipC1 and S19-3 also offered adaptive advantage over other genotypes under DS. Variation in intrinsic RLD in deeper soil depths in the studied genotypes determines root foraging capacity when facing DS. This suggests that different agroecological environments to which bambara groundnut is subjected in its natural habitat have promoted a phenotypic differentiation in root systems to adapt to ecotypic conditions, which may help offset the impact of DS. The natural genotypic variation exhibited, especially by DodR, could be exploited to identify potential quantitative trait loci (QTLs) that control deep rooting and root length density.

## Introduction

Drought is a major abiotic stress that lowers the yield of grain legume crops including bambara groundnut [*Vigna subterranea* (L.) Verdc]. Bambara groundnut is well-known for its ability to endure dry environments in comparison to other grain legumes, although adaptation mechanisms are unclear. In semi-arid Africa, it is third to groundnut and cowpea in terms of production and consumption (Sellschop, 1962). Bambara groundnut is set to increase in importance in marginal areas as production systems become more diverse to adapt to climate change (Mayes et al., 2012; Massawe et al., 2015; Feldman et al., 2019; Mustafa et al., 2019a,b). According to various climate models, many drought-stricken areas in eastern and southern Africa are expected to become drier in the coming decades (Rippke et al., 2016). To mitigate this and satisfy the growing demand for food amid population growth, efforts are underway to develop climate-resilient and nutritious crop varieties (Halimi et al., 2019; Mustafa et al., 2019a,b; Tan et al., 2020). In addition to genetics and genomics approaches, a detailed above-ground to below-ground phenotyping strategy in bambara groundnut breeding needs to be the focus.

Progress in drought phenotyping in bambara groundnut for the past 30 years has been elucidated by above ground shoot traits (Collinson et al., 1997, 1999; Jørgensen et al., 2010; Sesay et al., 2010; Vurayai et al., 2011a,b; Mabhaudhi and Modi, 2013; Chibarabada et al., 2015a,b; Chai et al., 2016a,b; Muhammad et al., 2016). This has proved fruitful, revealing the potential in selecting individual lines with improved drought resistance. However, less explored is the below ground root system architecture (RSA). RSA is an important developmental trait which plays a vital role in plant adaptation and productivity, especially under drought stress (DS) (Lynch, 2013). Indeed, bambara groundnut has evolved DS adaptation mechanisms under natural selection (Mateva et al., 2020). As a result, natural genotypic variation in RSA in bambara groundnut that is native to various agroecological conditions ranging from tropical wet to semiarid would be worthwhile to investigate.

However, the various interactions between RSA and different agroecological environments make it difficult to establish a root system ideotype that enhances both the capture of mobile water and nutrients (Lynch and Wojciechowski, 2015). For example, under drying soil conditions, there is generally an increase in root elongation (Raja and Bishnoi, 1990; Schmidhalter et al., 1998), but there is also evidence of reduced (King and Bush, 1985; Ogawa et al., 2005) and postponed (Bontpart et al., 2020) root length density (RLD). Moreover, when the soil surface slowly dries down, a reduction in RLD in deeper soil depths may aggravate the effects of water stress, triggering the reduction of essential processes such as stomatal conductance. This discrepancy might result from natural genotypic differences. For example, in consistently dry environments, bambara groundnut genotypes, such as S19-3 (a classic “drought escape-type” sourced from Namibia), possesses a quick-deep-cheap rooting system as opposed to a shallow-costly rooting system which is considered to be more beneficial in drought-prone regions (Mateva et al., 2020). As a result, uncovering and integrating such beneficial variants into new elite bambara groundnut varieties may be crucial in optimising efficiency and establishing plant ideotypes for drought environments.

In consistent agroecological environments, genotypic variation for root system traits has been shown to distinguish functional plant types (Leva et al., 2009). This has been included in crop breeding programmes that are oriented toward low-input systems in which the availability of soil resources are spatio-temporally dynamic (Lynch and Brown, 2012). However, Schneider and Lynch (2020) argue that an architectural model rigidly formed by the plant genome would make the root system poorly reactive, irrespective of the benefits of particular root traits, whereas root system developmental plasticity would be desirable in highly variable agroecological environments. Considering that plasticity is difficult to achieve due to the inability to reliably generate the best phenotype, evolving environmental signals, and/or the fact that phenotypic plasticity is expensive (Via and Lande, 1985), natural genotypic variation could be explored further in bambara groundnut. This could allow for genotypes to be assigned to specific agroecological environments and production systems.

Bambara groundnut is an interesting drought-tolerant crop for exploring such a line of inquiry in research. This grain legume flourishes under contrasted environments. Originating in West Africa, its distribution spans across aridity gradients from tropical dry climates in Senegal and Kenya, respectively, down to arid and semi-arid regions in sub-Saharan Africa. This is on soils which are poor in nutrients and are formed under variable pedoclimatic conditions. Indeed, farmers have grown bambara groundnut as genetically variable landraces for many centuries in the same agroecological climates. Comparing eight core parental lines of bambara groundnut — single genotypes derived from landraces of contrasting geographic origin — our previous studies found great variation in several shoot (Gao et al., 2020) and RSA traits (Mateva et al., 2020). As a provisional explanation, we hypothesise that farmers over the years may have indirectly selected for differences in the root system, particularly deep rooting and RLD in landraces from dry environments. This adaptation mechanism would be critical for plants exposed to DS, especially at the flowering stage when plants are most vulnerable to DS. In order to test these hypotheses, we compared rooting dynamics at two key stages, i.e., 55 days after emergence (DAE): 30 days of DS recovery and at 105 DAE: 50 days of DS recovery in eight bambara groundnut genotypes.

## Materials and Methods

### Polyvinyl Chloride Column System, Plant Material, and Growth Conditions

This work completes a previous study by Mateva et al. (2020) to which the reader is directed for a thorough explanation of the plant material and soil-filled polyvinyl chloride (PVC) column configuration. This study was conducted under a fixed rainout shelter during two consecutive seasons (2018 and 2019) at Crops for the Future (CFF) in Malaysia (2°55′52.2″N 101°52′45.7″E). Eight single genotypes were used to represent an aridity gradient (see Supplementary Figure 1). Gresik was sourced from Southeast Asia (humid and high rainfall habitat), while genotypes LunT, Ankpa-4, Tiga nicuru (West Africa), IITA-686, and DodR (East Africa) were from dry environments with rainfall > 570 per annum. Lastly, genotypes S19-3 and DipC1 were both from southern Africa (hot-dry habitat) (see Supplementary Figure 1).

The experiments consisted of a factorial treatment combination of eight genotypes (Gresik, LunT, IITA-686, DodR, S19-3, Tiga nicuru, Ankpa-4, and DipC1) and two water management (WM) treatments [well-watered, WW; and drought stress, DS; with three replicate plants per Genotype (G) × WM], giving a total of 96 individual plants. For the two water treatments, i.e., WW and DS, the PVC columns were slowly irrigated once every 3 days at 17.00 h to field capacity until 25 DAE. The irrigation was continued in the WW treatment right up to the end of the experiment, while the DS treatment received no further irrigation from 25 DAE (Supplementary Figure 2). Plants were subjected to DS for a period of 30 days. The DS treatment was terminated at 55 DAE (first destructive sample point), which also marked the end of the 30-day DS. Irrigation was resumed and all the plants, i.e., WW and DS (recovery), were slowly irrigated once on alternate days until final harvest (105 DAE; second destructive sample point; Supplementary Figure 2), which also marked 50-day of DS recovery. The soil substrate (sandy clay) was composed of silt (10%), sand (48%), clay (42%), 4.2% organic matter, 0.13% N, 41.0 (mg L^–1^) P, 0.16 (meq 100 g^–1^) K, 0.11 (meq 100 g^–1^) Mg, 1.09 (meq 100 g^–1^) Ca, 151.3 (mg L^–1^) Fe, and had a pH of 5.7 according to a soil test performed by Applied Agricultural Resources Sdn Bhd, Malaysia. Basal fertiliser, weed, and pest control were carried out as previously described [for more details see Mateva et al. (2020)]. Air temperature and relative humidity (RH) were monitored at 150 cm height level from the ground using Tip-Temp EL-USB-2-LCD thermo-hygrometers (Tip-Temperature, Burlington, NJ, United States). Daily air temperature values and RH were used to estimate the daily values of vapour pressure deficit (VPD).


(1) $$V\,P\,D\,\left(k\,P\,a\right)=\left(100-\frac{R\,H}{100}\right)\times \text{SVP}$$

where RH is relative humidity and SVP is the saturation vapour pressure calculated as previously described (Murray, 1967):


(2) $$S\,V\,P=\left[610.7\times \left(\frac{107.5\,\,\left(\text{T}\right)}{237.3+\left(\text{T}\right)}\right)\right]$$

where T is the air temperature.

### Root Traits

Root harvesting was conducted twice in each season: at 55 DAE (30 days of DS) and 105 DAE (50 days of DS recovery). Three biological replicates were used per treatment for root trait measurements per bambara groundnut genotype. Root samples were washed free of soil using a soft spray watering head. After complete removal of the soil, the shoots (i.e., leaves and stems) were separated from roots and entire root systems were placed in plastic zip lock bags and stored at 4°C. In order to identify and measure the taproot length (TRL cm plant^–1^), entire roots were laid flat and stretched against a 2-m ruler, giving an estimate of the deepest extent of the root system. Following Mateva et al. (2020), root systems were cut into 30 cm segments, representing different soil depths (i.e., 0–30, 30–60, 60–90, and 90–110 cm). Each root section was separately scanned in grayscale at 400 dots per inch using a flatbed Epson Scanner (Epson Perfection V700, Inc., Los Alamitos, CA, United States) with the WinRhizo Pro software v2009 (Regent Instruments, Montreal, QC, Canada). The root fresh weight (RFW) (g plant^–1^) were taken, and the RLD (cm cm^–3^) was calculated using the following equation:


(3) $$R\,L\,D=\left(\frac{R\,o\,o\,t\,l\,e\,n\,g\,t\,h}{S\,o\,i\,l\,v\,o\,l\,u\,m\,e}\right)$$

### Shoot Traits

At 55 DAE (30 days of DS) and 105 DAE (50 days of DS recovery), shoot height (SH) and number of leaves (NoL) were measured on a fresh plant basis. SH was recorded from the root crown to the apex of the longest plant stem using a ruler, whilst NoL was recorded as the number of fully expanded trifoliate leaves. Shoots were excised and partitioned into leaves and stems to weigh shoot fresh weight (SFW). Shoot and root materials were both oven-dried at 80°C for 72 h and then weighed for shoot dry weight (SDW) and root dry weight (RDW). Root to shoot (R:S) ratio was calculated on a dry mass basis by dividing RDW by SDW. Pods were dried and shelled, and the seeds were weighed to determine grain yield (g plant^–1^).

### Reproductive Development

Days to 50% flowering was recorded as the number of DAE when 50% of the plants had flowered.

### Stomatal Conductance

During DS, stomatal conductance (g_s_) was measured in a time-course experiment at 35, 45, and 55 DAE after water was withheld using a dynamic diffusion AP4 cycling porometer (Delta-T Devices Ltd, Cambridge, United Kingdom). Three biological replicates for g_s_ were taken between 11:00 h and 12 noon on the youngest, fully expanded trifoliate leaf. This was always done on a sunny day in Semenyih, Malaysia (average sunrise, sunset, and day length: 07.02, 19.15, and 12.11 h, respectively).

### Volumetric Water Content

Volumetric water content (VWC) was measured in a time-course experiment at 35, 45, and 55 DAE after water was withheld. Three biological replicates were measured using a handheld soil moisture ML2 Delta-T thetaProbe (ThetaProbe ML2, Delta-T Devices Ltd, Cambridge, United Kingdom) from the top part (every 30 cm) down to the bottom part of the soil profile via the pre-drilled holes in the sides of the PVC columns. Similar to g_s_, VWC readings were measured every week between 11:00 h and 12 noon during the WW and DS treatment periods, with measurements for the former performed before irrigating to avoid reading fluctuations.

### Statistical Analysis

Data on each measurable trait was subjected to two-way analysis of variance (ANOVA) to test the effects of the G and WM and their interaction (G × WM) using the Statistica Version 13.3 (TIBCO Software Inc., Palo Alto, CA, United States). Means were separated using Tukey’s Honestly Significant Difference (HSD) at the 5% level of significance. Linear equations and correlation coefficients were calculated with the SigmaPlot Version 12.5 software (Systat Software, Inc., San Jose, CA, United States).

## Results

### Plant Flowering

**FIGURE 1:**
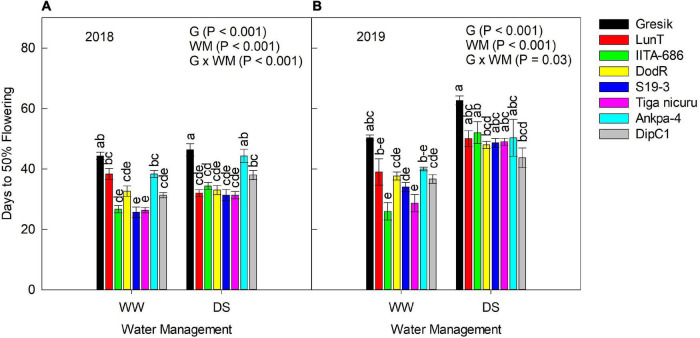
Interaction effect Genotype (G) × Water management (WM) on days to 50% flowering of eight bambara groundnut genotypes grown in a soil-filled polyvinyl chloride (PVC) columns in a rainout shelter **(A)** well-watered (WW) and drought stress (DS) during 2018, **(B)** WW and DS during 2019. The data is mean ± SE values (*n* = 3), with different letters showing significant differences by Tukey’s honest significant difference *post hoc* test for treatments.

According to the analysis of variance, days to 50% flowering was significantly affected by the interaction effect of genotype and WM for the 2018 (*p* < 0.001) season (Figures 1A,B). During the 2018 season, there was discrimination for days to 50% flowering between WW and DS treatments, with the latter showing longer days to 50% flowering values for most genotypes except for LunT. Based on the mean values for the genotype, days to 50% flowering for WW plants ranged from 26 (S19-3) to 44 days (Gresik), while that of the DS plants varied between 31 days (both S19-3 and Tiga nicuru) and 46 days (Gresik).

Similarly, in the 2019 season, days to 50% flowering was significantly affected by the interaction effect of genotype and WM (*p* = 0.03). However, in this particular season, IITA-686, S19-3, and Tiga nicuru showed significantly (*p* < 0.001) longer days to 50% flowering under DS in 2019 as compared to the WW values. Generally, the discrimination between WW and DS plants in 2019 was consistent with the 2018 season apart from LunT which flowered 6 days earlier in 2018. Based on mean values for the genotype selections in 2019, days to 50% flowering of the WW plants ranged from 26 (IITA-686) to 50 days (Gresik), while that of the DS-stressed plants varied from 44 (DipC1) to 63 days (Gresik).

### Plant Size and Number of Leaves

The interaction between genotypes and WM was not significant (*p* > 0.05) for SH and NoL at 55 DAE in both 2018 and 2019 seasons (Table 1). However, SH showed significant differences between genotypes (*p* < 0.01 and *p* < 0.001 in 2018 and 2019, respectively) and highly significant differences between WM (*p* < 0.001; for both seasons). In 2018, the genotypes DodR and Ankpa-4 (both 23.4 cm plant^–1^) showed significantly higher SH than Tiga nicuru (15.5 cm plant^–1^). In 2019, the genotype DipC1 (33.2 cm plant^–1^) showed significantly higher SH than Tiga nicuru (27.32 cm plant^–1^), Gresik (26.70 cm plant^–1^), and LunT (25.8 cm plant^–1^). Based on mean values for WM only, DS significantly reduced SH by 21 and 12% in the 2018 and 2019 seasons, respectively (Table 1).

**TABLE 1:** Analysis of variance (ANOVA) for shoot height (SH), and number of leaves (NoL) at 55 days after emergence (DAE) of eight bambara groundnut genotypes, grown in soil-filled polyvinyl chloride (PVC) columns in a rainout shelter under well-watered (WW) and drought stress (DS) in two seasons 2018 and 2019.

		SH		NoL^2^	
		(cm plant^–1^)		(number)	
Treatment^1^	*N*	55 DAE (2018)	55 DAE (2019)	55 DAE (2018)	55 DAE (2019)
**G**					
Gresik	6	19.62 ± 1.27^ab^	26.70 ± 1.46^b^	44 ± 9.46^a^	55 ± 10.41^a^
LunT	6	17.40 ± 0.88^ab^	25.81 ± 0.89^b^	20 ± 5.60^b^	33 ± 5.60^b^
IITA-686	6	22.92 ± 2.63^ab^	28.86 ± 0.56^ab^	30 ± 6.86^ab^	43 ± 6.86^ab^
DodR	6	23.40 ± 1.38^a^	30.87 ± 1.57^ab^	27 ± 5.92^ab^	40 ± 5.92^ab^
S19-3	6	17.62 ± 1.96^ab^	29.22 ± 0.95^ab^	28 ± 5.03^ab^	41 ± 5.03^ab^
Tiga nicuru	6	15.47 ± 1.25^b^	27.32 ± 2.30^b^	39 ± 7.71^ab^	52 ± 7.71^ab^
Ankpa-4	6	23.40 ± 3.11^a^	30.40 ± 1.79^ab^	38 ± 9.69^ab^	51 ± 9.69^ab^
DipC1	6	21.77 ± 1.72^ab^	33.19 ± 2.04^a^	32 ± 8.49^ab^	45 ± 8.49^ab^
**WM**					
**WW**	24	22.59 ± 1.00^a^	31.00 ± 0.86^a^	44 ± 3.56^a^	50 ± 3.13^a^
**DS**	24	17.81 ± 0.91^b^	27.10 ± 0.65^b^	19 ± 1.15^b^	30 ± 1.38^b^
**F probability**					
G		<0.01**	<0.01***	0.01**	0.02*
WM		<0.01***	<0.01***	<0.01***	<0.01***
G × WM		0.58^ns^	0.11^ns^	0.52^ns^	0.40^ns^

*^1^Treatments: G, genotype; WM, water management.*

*^2^NoL values rounded to the nearest integer because NoL represents discrete data.*

The data is mean ± SE values (n = 6), with different letters showing significant difference (HSD) as follows: *p < 0.05, **p < 0.01, and ***p < 0.001, and ns, not significant.

With respect to NoL at 55 DAE, there were significant differences between genotypes in 2018 and 2019 (*p* < 0.01 and *p* < 0.05, respectively), along with highly significant differences between WM (*p* < 0.001; for both seasons). Based on mean values for genotype selections only, NoL, in 2018, ranged from 20 (LunT) to 44 (Gresik). Similarly, in 2019 plants varied from 33 (LunT) to 55 (Gresik). Based on mean values for WM only, DS significantly reduced NoL by 57% in 2018 and 39% in 2019.

**FIGURE 2:**
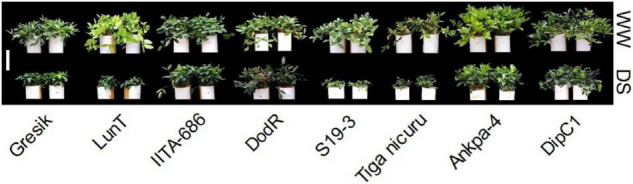
Differential plant shoot sizes at final harvest: 105 days after emergence (DAE; 50 days of DS recovery) of eight bambara groundnut genotypes grown in soil-filled PVC columns in a rainout shelter under WW and DS (at 50 days of recovery) in 2019. White bar = 30 cm.

Rewatering bambara groundnut plants after DS treatment resulted in a highly significant (*p* < 0.001) interaction effect between genotypes and WM for both SH and NoL at 105 DAE (50 days of DS recovery) in 2018 and 2019 seasons (Table 2 and Figure 2). In both seasons, SH was mostly lower in plants at 105 DAE (50 days of DS recovery) compared to WW, except for the genotype LunT which fully recovered and increased by 4% in DS (recovery) in 2018 and DodR (increased by 19%) in 2019. It is also worth noting that the genotypes DipC1, IITA-686, and DodR showed significant (*p* < 0.001) decrease in SH under DS (29, 25, and 25%, respectively) than in WW in the 2018 season, while other genotypes showed no significant difference between WW and DS (50 days of DS recovery). In 2019, LunT and S19-3 also showed a significant (*p* < 0.001) decrease in SH under DS (49 and 39%, respectively) than in WW.

**TABLE 2:** Analysis of variance (ANOVA) for SH, and NoL at 105 DAE of eight bambara groundnut genotypes, grown in soil-filled PVC columns in a rainout shelter under WW and DS in two seasons 2018 and 2019.

		SH		NoL^2^	
		(cm plant^–1^)		(number)	
Treatment^1^	N	105 DAE (2018)	105 DAE (2019)	105 DAE (2018)	105 DAE (2019)
**G × WM**					
**WW**					
Gresik	3	28.13 ± 0.61^abc^	37.15 ± 1.85^abc^	118 ± 6.24^b^	138 ± 7.75^b^
LunT	3	24.10 ± 1.07^cde^	43.98 ± 1.88^ab^	47 ± 12.58^cd^	67 ± 11.67^c–f^
IITA-686	3	32.83 ± 0.52^a^	39.56 ± 0.89^ab^	123 ± 26.12^b^	133 ± 14.45^b^
DodR	3	30.93 ± 0.93^ab^	34.37 ± 3.40^a–d^	81 ± 16.86^bc^	95 ± 13.02^bcd^
S19-3	3	22.43 ± 2.03^cde^	39.88 ± 2.44^ab^	46 ± 5.77^cd^	63 ± 2.60^def^
Tiga nicuru	3	23.07 ± 0.55^cde^	35.87 ± 1.13^a–d^	43 ± 6.66^cd^	59 ± 5.86^def^
Ankpa-4	3	26.10 ± 0.64^bcd^	48.56 ± 1.28^a^	225 ± 6.23^a^	219 ± 5.86^a^
DipC1	3	32.80 ± 1.86^a^	45.51 ± 2.23^ab^	63 ± 5.36^bcd^	99 ± 6.57^bcd^
**DS**					
Gresik	3	25.03 ± 1.93^b–e^	32.27 ± 2.34^bcd^	86 ± 3.06^bc^	99 ± 5.49^bcd^
LunT	3	25.03 ± 1.43^b–e^	22.34 ± 0.75^d^	20 ± 3.18^d^	34 ± 4.51^f^
IITA-686	3	24.60 ± 1.08^cde^	33.14 ± 2.73^bcd^	63 ± 8.84^bcd^	81 ± 9.49^cde^
DodR	3	23.33 ± 0.09^cde^	42.46 ± 2.39^ab^	50 ± 7.67^cd^	62 ± 8.19^def^
S19-3	3	19.93 ± 0.54^e^	24.42 ± 1.80^cd^	30 ± 0.33^cd^	44 ± 2.03*^ef^*
Tiga nicuru	3	21.60 ± 0.40^de^	24.40 ± 1.93 ^cd^	28 ± 2.19^cd^	37 ± 3.76^f^
Ankpa-4	3	21.03 ± 1.47^de^	39.04 ± 7.74^abc^	111 ± 24.38^b^	108 ± 13.20^bc^
DipC1	3	23.40 ± 0.82^cde^	35.58 ± 2.60^a–d^	82 ± 6.57^bc^	73 ± 2.33^c–f^
**F probability**					
G		<0.01***	<0.01***	<0.01***	<0.01***
WM		<0.01***	<0.01***	<0.01***	<0.01***
G × WM		<0.01***	<0.01***	<0.01***	<0.01***

*^1^Treatments: G, genotype; WM, water management.*

*^2^NoL values rounded to the nearest integer because NoL represents discrete data.*

The data is mean ± se values (n = 3), with different letters showing significant difference (HSD) as follows: ***p < 0.001, and ns, not significant.

Similarly, in both seasons, NoL was mostly lower in plants at 50 days of DS recovery compared to WW, except for the genotype DipC1 in the 2018 season which recorded 23% more NoL in the DS than the WW treatment (Table 2). The genotypes Ankpa-4 (2018 and 2019) and IITA-686 (2019) showed significant (*p* < 0.001) decrease in NoL under DS (51, 51, and 39%, respectively) than in WW.

### Root to Shoot Ratio

**FIGURE 3:**
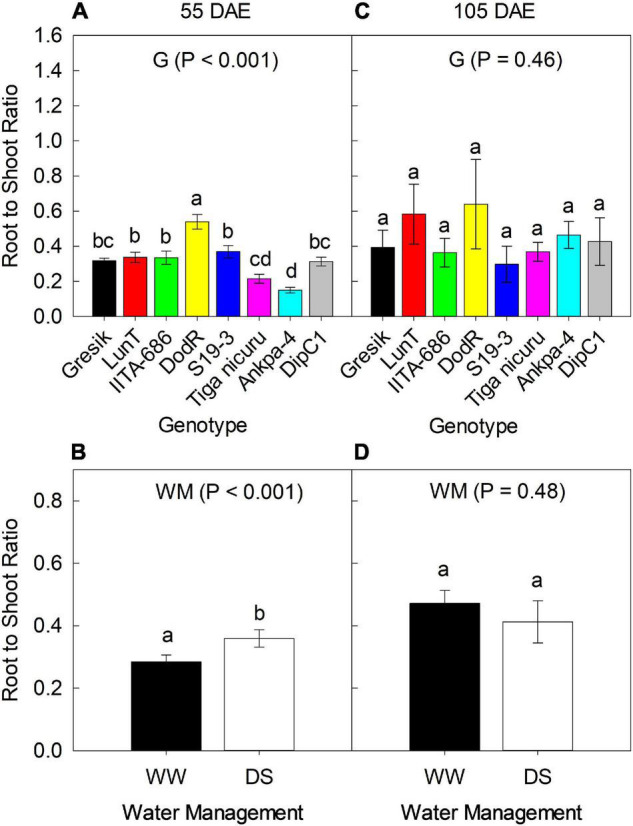
Effect of Genotype (G) — **(A,C)** at 55 and 105 DAE (50 days of DS recovery), respectively, the data is mean ± SE values (*n* = 6) and WM — **(B,D)** at 55 and 105 DAE (50 days of DS recovery), respectively, the data is mean ± SE values (*n* = 24) on root to shoot ratio (R:S) of eight bambara groundnut genotypes during the 2018 season. Different letters showing significant differences by Tukey’s honest significant difference *post hoc* test for treatments.

**FIGURE 4:**
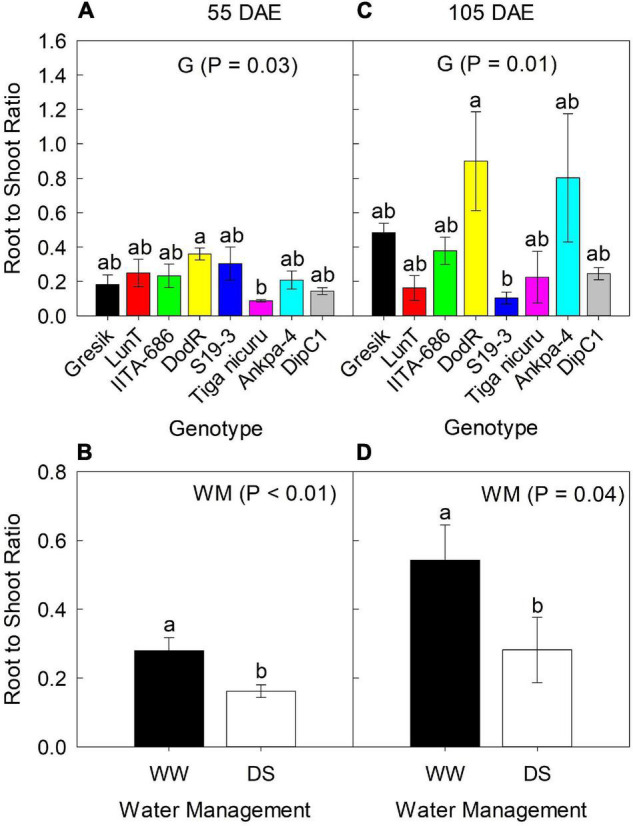
Effect of Genotype (G) — **(A,C)** at 55 and 105 DAE (50 days of DS recovery), respectively, the data is mean ± SE values (*n* = 6) and WM — **(B,D)** at 55 and 105 DAE (50 days of DS recovery), respectively, the data is mean ± SE values (*n* = 24) on root to shoot ratio R:S of eight bambara groundnut genotypes during the 2019 season. Different letters showing significant differences by Tukey’s honest significant difference *post hoc* test for treatments.

There was no significant interaction effect between genotypes and WM during 2018 and 2019 seasons with respect to R:S ratio at 55 and 105 DAE (50 days of DS recovery) (Figures 3, 4). Results of R:S ratio at 55 DAE for the 2018 season showed significant differences (*p* < 0.001) between the genotypes. The eight genotypes showed substantial differences in biomass allocation with R:S ratio ranging from 0.15 (Ankpa-4) to 0.54 (DodR) in 2018 (Figure 3A). Differences in WM were also observed with R:S ratio to be significantly higher (*p* < 0.001; 22%) under DS in 2018 compared to WW (Figure 3B).

Despite the lack of statistical difference among genotypes (*p* = 0.46; Figure 3C) at 105 DAE (50 days of DS recovery) in the 2018 season, R:S ratio was lower in the genotype IITA-686 (0.36) and highest in DodR (0.62) which was only 6% more than the second highest LunT (0.58). Differences in WM revealed higher R:S ratio in WW, although this was not statistically different (*p* = 0.48; Figure 3D) from the DS treatment.

Results of R:S ratio at 55 DAE for the 2019 season showed significant differences (*p* = 0.03) between the genotypes, ranging from 0.09 (Tiga nicuru) to 0.36 (DodR; Figure 4A). R:S ratio was significantly higher (*p* < 0.01; 42%) under WW compared to DS in the same season (Figure 4B). At 105 DAE (50-day of DS recovery) in the 2019 season, R:S ratio ranged from 0.10 (S19-3) to 0.90 (DodR; Figure 4C), with significantly higher (*p* = 0.04; 48%) R:S ratio under the WW treatment in 2019 compared to DS (Figure 4D).

### Changes in Root Depth Profile

**FIGURE 5:**
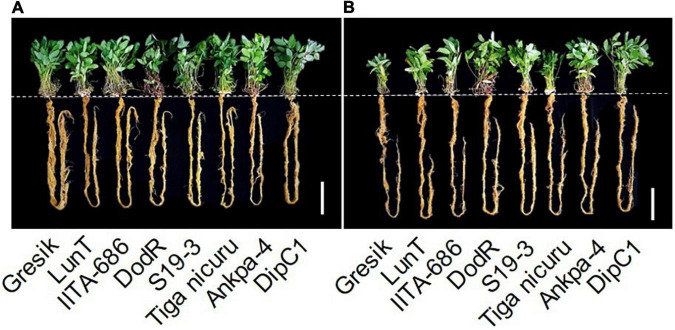
Example images of eight bambara groundnut genotypes grown in soil-filled PVC columns in a rainout shelter under **(A)** WW and **(B)** DS treatment at 55 DAE during 2019. White bar = 15 cm.

Root depth profile, i.e., TRL was significantly affected by the interaction effect of genotype and WM at 55 DAE for the 2018 season (*p* < 0.01; Table 3). However, TRL was not significantly affected by the interaction effect of genotype and WM at 55 DAE for the 2019 season (*p* = 0.94; Table 3 and Figure 5). TRL showed a significant decrease (*p* < 0.001) under the DS treatment by 14 and 22% in 2018 and 2019, respectively. Based on mean values for the genotypes at 55 DAE in the 2018 season, LunT and IITA-686 showed significant (*p* < 0.001) decrease in TRL under DS (27 and 25%, respectively) than in WW, while DodR recorded an increase (1%) in TRL under DS.

**TABLE 3:** Analysis of variance (ANOVA) for tap root length (TRL) at 55 and 105 DAE (50 days of DS recovery) of eight bambara groundnut genotypes, grown in soil-filled PVC columns under a rainout shelter under WW and DS in two seasons 2018 and 2019.

		TRL (cm)			
Treatment^1^	N	55 DAE (2018)	55 DAE (2019)	105 DAE (2018)	105 DAE (2019)
**G**					
Gresik	6	93.30 ± 3.60^c^	85.58 ± 11.03^b^	100.76 ± 3.58	138.20 ± 2.50^ab^
LunT	6	80.53 ± 5.62^d^	86.19 ± 8.69^b^	97.89 ± 2.78	118.40 ± 10.70^b^
IITA-686	6	100.04 ± 6.61^abc^	111.04 ± 7.80^ab^	107.71 ± 2.98	121.90 ± 4.80^ab^
DodR	6	107.09 ± 2.86^ab^	117.76 ± 6.29^a^	109.02 ± 2.69	140.90 ± 3.30^a^
S19-3	6	100.76 ± 4.44^abc^	113.52 ± 6.52^a^	102.09 ± 4.94	124.00 ± 3.90^ab^
Tiga nicuru	6	95.77 ± 3.90^bc^	105.33 ± 4.25^ab^	102.35 ± 3.17	120.60 ± 11.50^ab^
Ankpa-4	6	106.32 ± 3.74^ab^	92.52 ± 8.77^ab^	109.50 ± 5.44	137.05 ± 2.10^ab^
DipC1	6	108.25 ± 4.61^a^	104.37 ± 7.66^ab^	108.22 ± 4.61	124.00 ± 2.50^ab^
**WM**					
WW	24	106.66 ± 1.76^a^	114.56 ± 3.38^a^	107.52 ± 2.11^a^	135.90 ± 2.10^a^
DS	24	91.35 ± 2.70^b^	89.52 ± 3.69^b^	101.86 ± 1.72^b^	120.50 ± 3.70^b^
G*WM					
**WW**					
Gresik	3	100.94 ± 1.60^a–d^	92.74 ± 5.66^a^	105.21 ± 6.62^a^	140.30 ± 2.80^a^
LunT	3	93.10 ± 0.24^bcd^	102.67 ± 4.81^a^	95.00 ± 2.12^a^	138.00 ± 3.80^a^
IITA-686	3	114.06 ± 2.99^a^	123.72 ± 11.68^a^	114.00 ± 1.99^a^	121.80 ± 10.60^ab^
DodR	3	106.50 ± 5.51^abc^	130.19 ± 5.17^a^	112.50 ± 3.34^a^	147.00 ± 3.90^a^
S19-3	3	108.87 ± 3.73^ab^	125.94 ± 6.27^a^	109.65 ± 7.82^a^	131.60 ± 3.10^ab^
Tiga nicuru	3	103.20 ± 1.49^a–d^	113.98 ± 2.75^a^	104.70 ± 5.10^a^	141.30 ± 2.20^a^
Ankpa-4	3	109.56 ± 4.08^ab^	106.46 ± 13.72^a^	108.32 ± 7.47^a^	138.70 ± 1.60^a^
DipC1	3	117.07 ± 2.85^a^	120.77 ± 2.46^a^	110.78 ± 8.86^a^	128.70 ± 2.90^ab^
**DS**					
Gresik	3	85.66 ± 1.92^de^	78.43 ± 22.91^a^	96.31 ± 0.69^a^	136.10 ± 4.40^a^
LunT	3	67.96 ± 0.29^e^	69.72 ± 9.11^a^	100.78 ± 5.07^a^	98.70 ± 13.10^b^
IITA-686	3	86.03 ± 3.65^de^	98.36 ± 2.66^a^	101.41 ± 0.91^a^	122.00 ± 1.70^ab^
DodR	3	107.68 ± 3.18^ab^	105.33 ± 4.10^a^	105.55 ± 3.60^a^	134.80 ± 1.40 ^a^
S19-3	3	92.66 ± 4.35^bcd^	101.09 ± 4.35^a^	94.52 ± 1.91^a^	116.40 ± 3.10^ab^
Tiga nicuru	3	88.34 ± 4.33^cd^	96.68 ± 2.84^a^	100.00 ± 4.34^a^	100.00 ± 15.20^b^
Ankpa-4	3	103.08 ± 6.53^a–d^	78.58 ± 1.34^a^	110.68 ± 9.53^a^	136.30 ± 4.30^a^
DipC1	3	99.43 ± 4.49^a–d^	87.98 ± 4.28^a^	105.66 ± 4.62^a^	119.40 ± 0.50^ab^
**F probability**					
G		<0.01***	0.01*	0.25^ns^	<0.01***
WM		<0.01***	<0.01***	0.04*	<0.01***
G*WM		0.01*	0.94^ns^	0.55^ns^	0.01*

*^1^Treatments: G, genotype; WM, water management.*

The data is mean ± SE values with different letters showing significant difference (HSD) as follows: *p < 0.05 and ***p < 0.001, and ns, not significant.

At 55 DAE in the 2019 season, TRL under DS ranged from 85.6 (Gresik) to 117.1 cm (DodR), with an average TRL of 102 cm (Table 3). Compared to DodR, the genotypes Gresik and LunT showed significantly (*p* < 0.05) less TRL which was exclusively limited to the 60–90 cm layer. The genotypes S19-3 showed significantly higher TRL, recording the second largest TRL (113.5 cm), although S19-3 was only 4 cm shorter than the deepest rooting DodR genotype.

For TRL at 105 DAE (50 days of DS recovery), no interaction effect (*p* = 0.55) and genotype effects (*p* = 0.25) were observed in the 2018 season (Table 3). TRL showed a significant decrease under the DS treatment by 5 and 11% in 2018 and 2019, respectively. Genotypes Tiga nicuru and LunT showed highly significant (*p* < 0.001) decreases in TRL under DS (29 and 28%, respectively) than in WW in the 2019 season, with IITA-686, Ankpa-4, Gresik, and DodR recording the least differences (1, 2, 3, and 8%, respectively) under DS (50-day of recovery).

### Changes in Vertical Root Distribution

**FIGURE 6:**
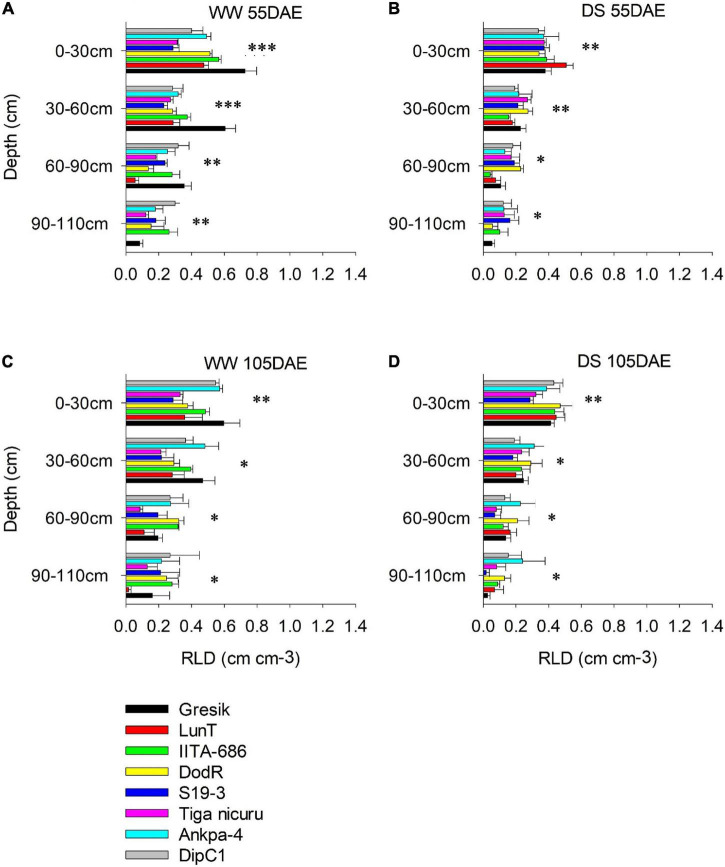
Root length density (RLD) of eight bambara groundnut genotypes at different soil depths grown in soil-filled PVC columns in the 2018 season **(A,B)** WW and DS at 55 DAE, respectively. **(C,D)** WW and DS at 105 DAE, respectively. Mean ± SE values (*n* = 3) are shown. Significant differences as follows: *p < 0.05, ^**^p < 0.01, and ^***^p < 0.001; and ns, not significant, among individual genotypes.

**FIGURE 7:**
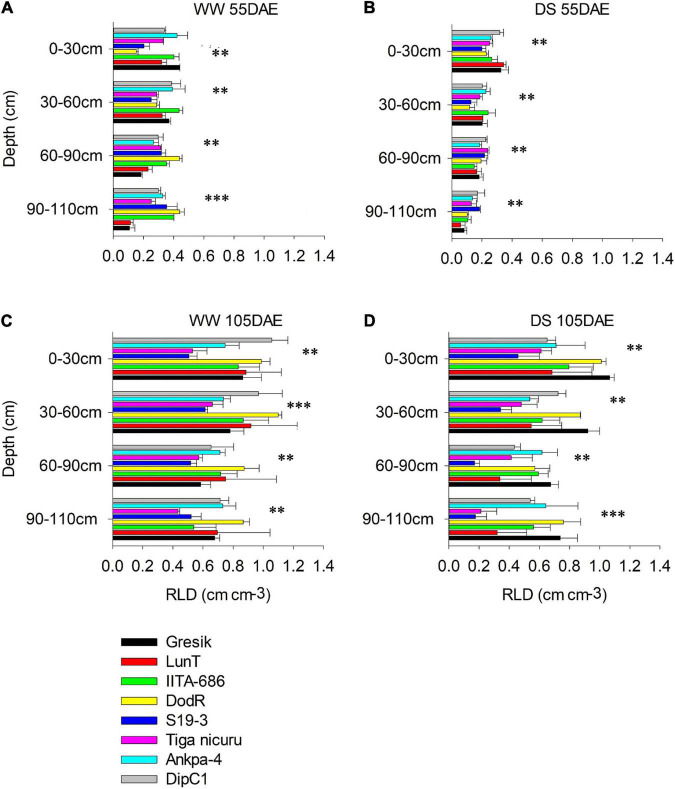
Root length density of eight bambara groundnut genotypes at different soil depths grown in soil-filled PVC columns in the 2019 season **(A,B)** WW and DS at 55 DAE, respectively. **(C,D)** WW and DS at 105 DAE, respectively. Mean ± SE values (*n* = 3) are shown. Significant differences as follows: ^**^p < 0.01 and ^***^p < 0.001; and ns, not significant, amongst individual genotypes.

Vertical root distribution, i.e., RLD (cm cm^–3^) was measured at various soil depths (i.e., 0–30, 30–60, 60–90, and 90–110 cm) and generally showed a decrease with soil depth. Within these soil depths, RLD at 55 DAE was significantly affected by the interaction effect of genotype and WM (*p* < 0.001; Figure 6) except at 90–110 cm (*p* = 0.41) in the 2018 season (*p* < 0.001; Figure 7A) and 30–60 cm (*p* = 0.88; Figures 7A,B) of soil depth in the 2019 season. RLD generally showed maximum distribution in the shallow 0-30 cm soil depth for most of the studied genotypes under both WW and DS treatment at 55 DAE in both 2018 and 2019 seasons (Figures 6A,B, 7A,B).

Under the WW treatment, the rainy-habitat genotype Gresik recorded the highest mean at the depth of 0–30 cm, with 0.73, and 0.44 cm cm^–3^ in the 2018 and 2019 seasons, respectively (Figures 6A, 7A). Under the DS treatment, RLD was reduced in almost all soil depths and all genotypes (Figures 6B, 7B). RLD was lower in the deeper soil depths, i.e., 60–90 and 90–110 cm, with the highest reduction observed in the 90–110 cm layer (42 and 58%) in the 2018 and 2019 seasons, respectively. Deeper soil depths revealed marked differences among genotypes with rainy-habitat genotype LunT having no roots in the 90- to 110-cm soil depth in 2018 and recording the least RLD (0.06 ± 0.013 cm cm^–3^) in 2019, whereas RLD of rainy habitat Gresik decreased progressively down to 110 cm in both seasons. Dry-habitat S19-3 densely occupied the soil at depth, retaining a RLD of 0.16 ± 0.05 and 0.18 ± 0.01 cm cm^–3^ in the deepest layer in both seasons, respectively (Figures 6B, 7A).

In the absence of stress, all plants recovered to a similar degree as shown by the consistent lack of a significant interaction effect of genotype and WM (*p* > 0.05) at 105 DAE (50 days of DS recovery) in both seasons in all soil depths (Figures 6C,D, 7C,D). However, significant differences were noted among genotypes in the 0-30, 30-60, and 60-90 cm soil depths (*p* < 0.001; *p* < 0.05; and *p* < 0.05, respectively) and WM in the 30–60, 60–90, and 90–110 cm soil depths (*p* < 0.001; *p* < 0.001; and *p* < 0.05, respectively) in the 2018 season. For the 2019 season, significant differences were noted among genotype selections in the 0–30, 30–60, and 90–110 soil depths (*p* < 0.001; *p* < 0.001; and *p* < 0.05, respectively) and WM in the 30–60, 60–90, and 90–110 cm soil depths (*p* < 0.001; *p* < 0.001; and *p* < 0.05, respectively). The reduced RLD under DS had a tendency to recover when re-watered. Genotypes LunT and DodR (0–30 cm), Tiga nicuru (30–60 cm), LunT (60–90 cm), and LunT and Ankpa-4 (90–110 cm) maintained high RLD under drought recovery, surpassing the density in the WW treatment in 2018 (Figures 6C,D). In the 2019 season, Gresik under DS surpassed the RLD in the WW treatment for all the soil depths. Recovery was also observed in Tiga nicuru and DodR (0-30 cm) and IITA-686 (90-110 cm; Figures 7C,D).

### Soil Moisture Content and Stomatal Conductance

**FIGURE 8:**
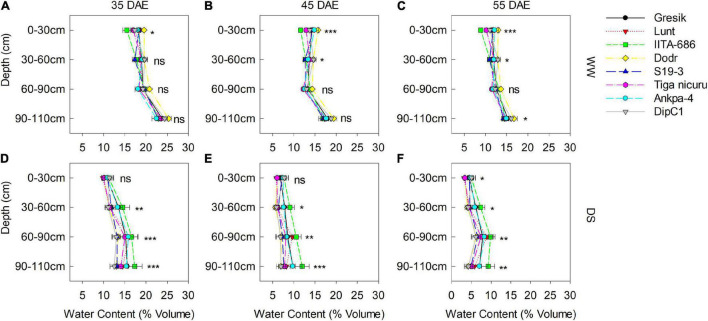
Soil volumetric water content measured in the soil-filled PVC columns under a rainout shelter at three time points (35, 45, and 55 DAE) in 2019. Measurements are of eight bambara groundnut genotypes grown under WW **(A–C)** and DS **(D–F)** treatments. Mean ± SE values (*n* = 3) are shown. Significant differences as follows: *p < 0.05, **p < 0.01, and ***p < 0.001; and ns, not significant, amongst individual genotypes.

**FIGURE 9:**
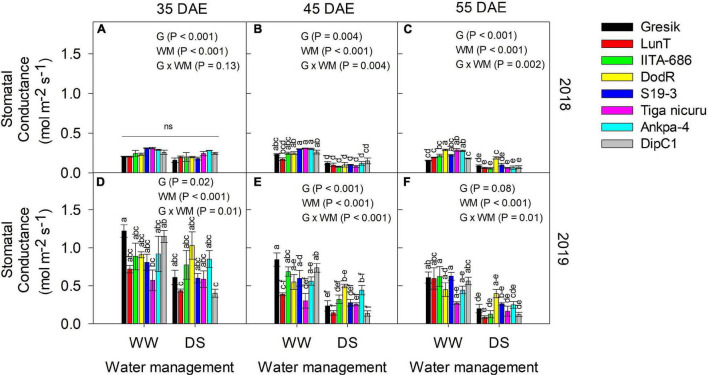
Interaction effect Genotype (G) × Water management (WM) on stomatal conductance, g_s_ (mol m^–2^ s^–1^) of eight bambara groundnut genotypes grown in soil-filled PVC columns under a rainout shelter at **(A–C)** 35, 45, and 55 DAE, respectively in 2018 and **(D–F)** 35, 45, and 55 DAE, respectively in 2019. The data is mean ± SE values (*n* = 3), with different letters showing significant differences by Tukey’s honest significant difference *post hoc* test for treatment with ns, not significant.

Volumetric water content (VWC) was only measured in 2019 at three time points, i.e., 35, 45, and 55 DAE (Figure 8). At 35 DAE, this averaged at 0.17 and 0.10 m^3^ m^–3^ in the surface soil (0–30 cm) for WW and DS (Figures 8A,D), respectively, while averaged 0.18 and 0.12 m^3^m^–3^ at 30–60 cm depth. Subsequent soil depths, i.e., 60–90 and 90–110 cm averaged 0.19 and 0.14 m^3^ m^–3^ and 0.23 and 0.14 m^3^ m^–3^ for WW and DS, respectively. At 45 DAE, VWC averaged 0.13 and 0.06 m^3^ m^–3^ in the surface soil (0–30 cm) for WW and DS (Figures 8B,E), respectively, while it averaged 0.13 and 0.06 m^3^ m^–3^ at 30–60 cm depth. Subsequent soil depths, i.e., 60–90 and 90–110 cm, respectively, averaged 0.13 and 0.08 m^3^ m^–3^ and 0.17 and 0.08 m^3^ m^–3^. Soil dried substantially by 55 DAE, with VWC dropping to 0.11 and 0.04 m^3^ m^–3^ in the surface soil (0–30 cm) for WW and DS (Figures 8C,F), respectively, while this in turn averaged 0.11 and 0.05 m^3^ m^–3^ at 30–60 cm depth. Subsequent soil depths, i.e., 60–90 and 90–110 cm averaged 0.12 and 0.07 m^3^ m–^3^ and 0.15 and 0.05 m^3^ m^–3^, respectively. The pattern of soil moisture depletion in the PVC columns was similar to the changes in stomatal conductance (g_s_) (Figure 9).

Stomatal conductance (g_s_) was measured in both the 2018 (Figures 9A–C) and 2019 (Figures 9D–F) seasons at three time points, i.e., 35, 45, and 55 DAE. The effect of water deficit stress on plants was determined by g_s_. Significant interaction effects of G and WM were observed across the three time points for both seasons, except at 35 DAE in 2018 (*p* = 0.13; Figure 9A). At this time point, significant differences were noted among G selections and WM (both *p* < 0.001; Supplementary Figure 3). In 2018, DS generally decreased g_s_ by 17, 59, and 73% at 35, 45, and 55 DAE, respectively (Figures 9A–C), whilst a 26, 51, and 68% decrease was observed in 2019 (Figures 9D–F). The eight genotypes at 55 DAE varied the most for g_s_ under DS in both seasons (2018 and 2019), ranging from 0.06 (IITA-686) to 0.10 mol m^–2^ s^–1^ (DodR) and 0.09 (LunT) to 0.25 mol m^–2^ s^–1^ (DodR), respectively (Figures 9C,F).

### Grain Yield

**FIGURE 10:**
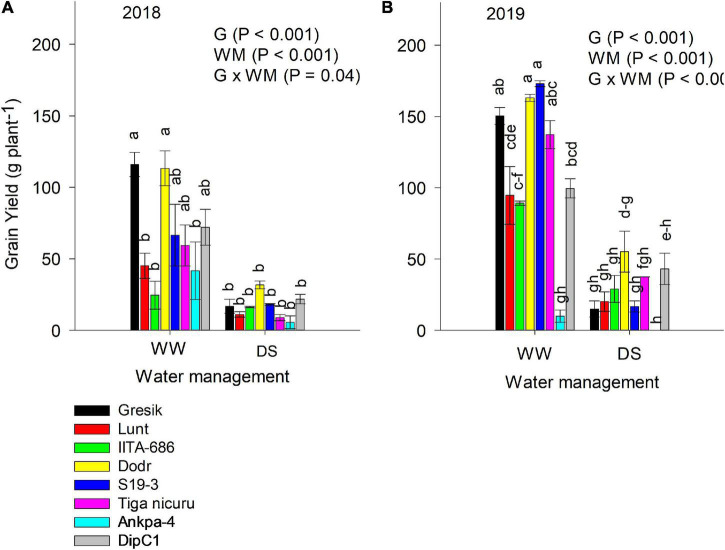
ANOVA for grain yield (g plant^–1^) in two seasons **(A)** 2018 and **(B)** 2019 under WW and DS. The data is mean ± SE values (*n* = 3) with different letters showing significant differences by Tukey’s honest significant difference *post hoc* test for treatments.

Significant interaction effects (*p* = 0.04; Figure 10A) between genotype and WM were observed in the 2018 season and a highly significant interaction (*p* < 0.001; Figure 10B) was observed in the following season (2019). Under the DS treatment, grain yield decreased by 76% in both the 2018 and 2019 seasons (Figure 10). IITA-686, DipC1, and DodR recorded the lowest reduction in grain yield in the 2018 season, whilst DipC1 and DodR had the lowest reduction in 2019. The genotype DodR was able to constantly produce the high grain yield under DS (31.7 g plant^–1^) in 2018 and (55.2 g plant^–1^) in 2019.

### Correlations Between Root Traits and Grain Yield Under Drought Stress and Well-Watered Treatment

**FIGURE 11:**
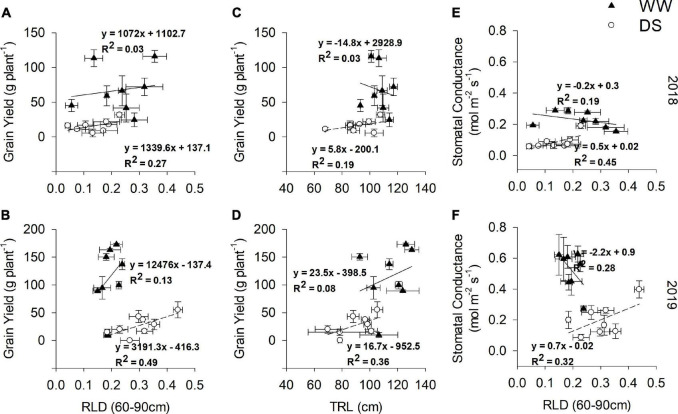
Relationship between RLD (60–90 cm) and grain yield (g plant^–1^), tap root length (TRL cm) and grain yield (g plant^–1^), and root length density (RLD 60–90 cm) and g_s_ at 105 DAE, for two seasons: 2018 **(A,C,E)** and 2019 **(B,D,F)** under WW and DS. Coefficient of determination *R*^2^ reported upon fitting with equation *y* = a*x + y0.

Significant negative correlations (*p* < 0.05; Supplementary Figure 5) between topsoil RLD (0–30 cm) at 55 DAE and grain yield were observed in the DS treatments in the 2018 and 2019 seasons (*R*^2^ = 0.20 and 0.31, respectively). In contrast to the topsoil, subsoil RLD (60–90 cm) at 55 DAE had weak (*R*^2^ = 0.27) and moderate (*R*^2^ = 0.49) positive correlations (*p* < 0.05; Figures 11A,B) with grain yield in the DS treatments in the 2018 and 2019 seasons, respectively. Significant positive correlations (*p* < 0.05; Figures 11C,D) were also observed between TRL at 55 DAE and grain yield in the 2018 and 2019 seasons (*R*^2^ = 0.19 and 0.36, respectively). The correlations between g_s_ and RLD (60–90 cm), both at 55 DAE, were positive and significant (*p* < 0.05) under DS treatment (*R*^2^ = 0.45 and *R*^2^ = 0.32 for 2018 and 2019 seasons, respectively), with negative correlations under the WW treatment (*R*^2^ = 0.19 and *R*^2^ = 0.28 for 2018 and 2019 seasons, respectively) (Figures 11E,F). Under the DS treatment, the genotypes DodR, S19-3, DipC1, and Tiga nicuru with high RLD (60–90 cm) at 55 DAE in 2018 were also strongly associated with high g_s_ at the same stage, i.e., 55 DAE (Figures 11E,F). In 2019, a somewhat similar trend was observed, with genotypes DodR, IITA-686, S19-3, and Tiga nicuru strongly associated with high g_s_ at 55 DAE (Figures 11E,F).

Shoot height (SH) was closely and positively correlated (*p* < 0.05) with RLD at 55 DAE in the deep 60–90 cm of the soil in both WW (*R*^2^ = 0.22 and *R*^2^ = 0.46 for 2018 and 2019 seasons, respectively) and DS treatments (*R*^2^ = 0.08 and *R*^2^ = 0.43 for 2018 and 2019 seasons, respectively; Supplementary Figure 4).

## Discussion

This research builds on a prior study by Mateva et al. (2020), which identified the presence of RSA variation in eight non-stressed bambara groundnut core parental lines, i.e., single genotypes derived from landraces from various agroecologies. These findings corroborated prior physiological evaluation (Mwale et al., 2007; Jørgensen et al., 2010, 2012; Berchie et al., 2012; Mabhaudhi and Modi, 2013; Mayes et al., 2013; Al Shareef et al., 2014; Chibarabada et al., 2015a,b; Chai et al., 2016b; Nautiyal et al., 2017) which had been largely limited to aboveground phenotyping. The present results show strong genotype-specific differences in root morphology in response to DS within the eight bambara groundnut core parental lines. Moreover, the genotype-specific differences were consistent with previous knowledge on the stomatal response of bambara groundnut to DS conditions (Jørgensen et al., 2010; Chai et al., 2016b). This study found enough differences to unequivocally identify the eight genotypes by their roots. In agreement with Serraj et al. (2004), Kashiwagi et al. (2006), Lalitha et al. (2015), and Mateva et al. (2020), the most critical morphological features were (i) root depth profile, i.e., TRL, which defines the soil volume that is exploitable, and (ii) the vertical root distribution, i.e., the root length distribution which regulates the effective capacity of foraging the soil volume.

### Bambara Groundnut Core Parental Lines as Models for Natural Variation of Root System Architecture

Core parental lines, i.e., single genotypes derived from landraces of contrasting geographic origin, have been established for bambara groundnut. Landraces from multiple ecosystems may be helpful for increasing the range of production zones because they have become adapted to their local environments as a result of continuous growth and selection in the same location (Mayes et al., 2012). Therefore, the availability of core parental lines (i.e., Gresik, LunT, IITA-686, DodR, S19-3, Tiga nicuru, Ankpa-4, DipC1) and their judicious use will be critical for breeding and selection programmes. Core parental lines are an ideal resource to identify new sources of variation. For example, substantial variation in photoperiodic effect has been reported in the core parental lines of bambara groundnut (Kendabie et al., 2020), whereas broad diversity in drought and heat tolerance (Dhanaraj, 2018; Rahmah et al., 2020) and phenotypic variability have been found (Gao et al., 2020). Clearly, the success of bambara groundnut drought breeding programmes is dependent on the extent of phenotypic variation present in the germplasm base. The ability to classify plant roots in these core parental lines (as demonstrated in Mateva et al., 2020) allows for more in-depth research for resilience to localized stresses.

Most bambara groundnut experiments have extensive aboveground trait phenotyping (Collinson et al., 1997, 1999; Jørgensen et al., 2010; Sesay et al., 2010; Vurayai et al., 2011a,b; Mabhaudhi and Modi, 2013; Chibarabada et al., 2015b; Chai et al., 2016a,b; Muhammad et al., 2016). Below ground biomass, on the other hand, is aggregated into a single, black-box group, and key questions to do with adaptation to water deficit stress remain unanswered. To observe bambara groundnut root development, plants were grown in low-cost vertically oriented lightweight PVC columns filled with soil. Certain aspects of the research methodology adapted in the present study aided in the identification of genotypes based on their origin. For example, because of the sandy clay classification of the soil, it was possible to extract and clean the individual root depth segments with little alteration to their morphology. A gentle wash of the roots separated them from all soil particles clinging to them. Moreso, with this low-cost PVC column phenotyping system and image analysis set-up, identification of variation in root depth profile and vertical root distribution was made possible, indicating inherent natural phenotypic diversity and the potential of the core parental germplasm collection to reveal quantitative trait loci (QTLs) involved in root development.

### Reaching the Soil at Depth by Tap Root Length

In conditions of periodic drought, bambara groundnut is planted in moist soil either after or intercropped with major cereal crops (Chibarabada et al., 2015b). Due to drainage, evaporation, and plant water intake, the soil gradually dries from the surface, resulting in substantially increased water availability in deeper soil layers and progressively harder top soils (Lynch, 2013). This dry-down scenario delays flowering, leading to a decrease in grain yield (Pang et al., 2017). Bambara groundnut has to quickly develop a deep tap root system in order to explore deeper soil depths before surface soil layers dry out (Wasaya et al., 2018). Failure to do so minimises the ability to forage for stored deep soil moisture reserves which accumulate at the beginning of the rainy season. Results from a previous analysis of TRL at the pre-flowering (35 DAE) stage (Mateva et al., 2020) showed average values of nearly 92 cm as early as 35 DAE in the tap root of hot dry-habitat S19-3, DipC1 (both from southern Africa), dry-habitat DodR (east Africa), and Ankpa-4 (west Africa) against an average of nearly 66 cm in the other four genotypes that are mostly sourced from rainy habitats. Indeed, the present study also showed differential TRL among genotypes at 55 DAE (i.e., at the end of a 30-day drought stress).

Drought stress generally decreased TRL among the studied genotypes, but it did not decrease in the genotype DodR (from Tanzania: tropical dry climatic conditions). Previous research has shown that DS decreases root length (Avramova et al., 2016; Durand et al., 2016), root biomass (Price et al., 2002), and RLD (Fang et al., 2017) in a variety of plant species. In the present study, maintenance of TRL with an increase in the RLD in the deeper soil depth, i.e., 60–90 cm under DS treatment was observed in DodR. This, therefore, enabled consistent water foraging under dry soil conditions. Not only did DodR record the highest value for TRL as early as 55 DAE, but it also demonstrated an intrinsic ability for early flowering about 3 and 2 days earlier than mean flowering (36 and 50 days) time in both 2018 and 2019 study seasons, respectively. The genotype DodR from arid areas of Tanzania is well-suited to dry environments because of its capacity to penetrate and extract available water from deep within the soil profile. Similar results were observed in dry-habitat DipC1, with early flowering in the DS treatment in 2019 basically providing two critical advantages, i.e., low level-stress facilitating an extended reproductive duration and a better soil water availability and foraging which supported rapid rate in partitioning to grains (Figure 10B; Krishnamurthy et al., 2013; Purushothaman et al., 2014). Several studies confirm that drought can induce plants to develop a deeper TRL as an adaptive response (Gregory, 2006; Rellán-Álvarez et al., 2015; Wasaya et al., 2018). In the present study, an increase in TRL allowed the DodR plants to compensate for the gradually declining soil water availability by quickly exploring a much greater volume of soil as demonstrated by continued reduction of lower soil VWC values and maintenance of stomatal conductance (Figure 8F). Moreover, the RLD in deeper soil depths showed that this was indeed the case (Figure 6), which gave DodR, DipC1, and S19-3 a marked adaptive advantage over other genotypes. Therefore, these genotypes responded partly through drought escape and drought avoidance and remained stable across seasons. These adaptation mechanisms explain genotype-dependent adaptation to the different agroecologies they were sourced from.

### Foraging the Soil Volume by Root Length Density

Root branching density (BD) and branching intensity (BI) traits have a strong impact on water uptake. In this study, both traits were not measured. In fact, although useful, these traits can quickly become difficult to quantify as the plant nears maturity. In such cases, RLD is a useful trait that can be used as a proxy to estimate both root BD and BI (Mateva et al., 2020). For successful plant establishment, not only is a quick TRL of major importance, but a high RLD in deeper soil depths is also considered an adaptive root trait (Kashiwagi et al., 2005; Palta et al., 2011). The present study showed that RLD was significantly reduced in some of the studied genotypes, although to a lesser extent than aboveground shoot biomass. This resulted in increased differences among genotypes for R:S ratio. RLD was less affected by DS in rainy habitat Tiga nicuru and LunT and dry-habitat S19-3 than in the other five genotypes, with DodR and S19-3 maintaining the highest RLD and R:S ratio. RLD generally decreased the length of the soil column. The rainy habitat Gresik had the most RLD in the topsoil layer (0–30 cm) and used more water during the DS period (55 DAE), making less soil water available to plants after the flowering stage, which inevitably affected grain yield (Figure 10). Meanwhile, the lack of roots in the subsoil layer and inability to fully explore soil water in the deep layer may further explain why Gresik had the lowest grain yield in the 2019 season. The development and maintenance of root tissue require a substantial expenditure of resources (Nielsen et al., 2001). Early in plant development, the expenditure of carbon and nutrient resources in tissue construction and maintenance restricts the capacity to grow additional roots in various soil domains as resource availability changes. If roots proliferate early in the growth season in moist topsoil, for example, this decreases the potential for root development in deeper soil where resources are more likely to be found later in the season. Furthermore, early root proliferation in topsoil may not be useful later in the season in hard, dry surface soils. Passioura (1983) indicated that less RLD would be advantageous in the topsoil layer only if more water could be used in deep soil layers. On the other hand, it is worth noting that roots in topsoil often are involved in scavenging phosphorus (Lynch and Brown, 2001). Hence, adapted soil conditions may also be important. If the genotype Gresik were more adapted to low pH soils, then it may have been selected in P scavenging — not needing deep rooting. As a result, in low-input cropping systems, strategic recombination of P-efficient genotypes may increase crop productivity (Wafula et al., 2021). Also, while anecdotal, DodR was found to be performing well among several parental lines grown in waterlogged conditions during the rainy season in Indonesia, demonstrating the genotype’s robustness (Redjeki, personal communication, 2017).

Compared to the topsoil layer (0–30 cm) of the column, RLD in deeper soil depths (60–90 cm) gave a substantial positive contribution to grain yield. This contribution was highly consistent across the two seasons. Accordingly, RLD in the topsoil layer (0–30 cm) had a significant negative correlation with grain yield. These results are in agreement with Fang et al. (2011) who reported that greater RLD increases root competition and delays the effectiveness of roots in capturing water under DS conditions. In addition, this also aggravates abscisic acid (ABA) accumulation and subsequent stomatal closure (Tombesi et al., 2015). Previous work on wheat demonstrated that higher RLDs are critical for increased early vigour and pre-flowering water use, which would improve grain yield (Rebetzke and Richard, 1999). In our findings, DodR and S19-3 had relatively lower RLD in the topsoil layer compared to Gresik, but higher RLD in the subsoil layers in both seasons. This was positively associated with yield. Selecting for higher RLD in the subsoil layers is considered an option for the adaptation of wheat to water stress, increasing the water extraction capacity in the subsoil profile for grain filling and increased grain yield (Palta et al., 2011) especially under terminal DS (Passioura, 1983; Pooran et al., 2008). Angus and van Herwaarden (2001) argued that if subsoil water can be fully exploited between anthesis and grain filling, then grain yield will be significantly increased under drought stress. Looking at the deepest soil depth (90–110 cm), it is worth mentioning that the specific rooting pattern found in most dry-habitat genotypes might be due to an innate biological characteristic, but it could also be due to a methodological artefact. The majority of genotypes (especially at 105 DAE) reached the deepest soil depth (90–110 cm) and the physical constraint (i.e., detachable perforated plate) faced by the growing roots might have stimulated the development of new lateral roots. While this high RLD could be artefactual (Gregory, 2006), it still reveals the differential deep rooting vigour in the studied genotypes.

### Integrating Root System Architecture With Aboveground Plant Traits

Given that the root system is a hidden and complicated organ, the idea of indirect selection by utilising aboveground plant components seems quite appealing, although considerable errors have been reported (Casper and Jackson, 1997). In the present study, SH was positively correlated with the RLD in the 60–90 cm of the soil. A study by Mateva et al. (2020) also found that SH was closely and positively correlated with lateral branching in the deep 60–90 cm of the soil, and this was largely amongst genotypes originating from drier versus wetter agroecological environments. Furthermore, changes in root length has been observed in rice under water stress, and has been linked to increased shoot biomass and yield (Niones et al., 2013). The trend agrees with intensive studies on chickpea (Yinglong et al., 2017; Makonya et al., 2020) and wheat (Tolley and Mohammadi, 2020), which suggest that there is a persistent tendency of a positive correlation between roots and shoots. Since a plant is a biological entity, the root system absorbs water and nutrients for the stem and leaves, which then provide food for the root system’s maintenance. As a result, SH is a reasonable trait to consider as a proxy for estimating difficult-to-access vertical root distribution, especially when screening large populations. However additional genotypes would be needed to confirm this. Also, when mapped, traits with higher genotype-to-genotype correlations (such as RLD at 60–90 cm) are more likely to produce consistent QTLs.

## Conclusion

The present study is an initiative to better understand an ignored African grain legume with superior drought resistance relative to other cultivated grain legumes in Africa. To the best of our knowledge, we provide the first itemised report of deep rooting and RLD in core bambara groundnut parental lines subjected to a drought treatment. Differences in root system and shoot responses to a drought treatment were observed among the eight bambara groundnut genotypes due to their different genetic background and buffering capacity (i.e., plasticity related to G × W). These responses encompassed morphological root and shoot traits, such as TRL, RLD, SH, and NoL, in response to the DS treatment. These inherent characteristics govern root architecture and foraging dynamics, and hence have a direct impact on root system functionality. The present study found that on the basis of the closeness of their association with grain yield, drought resistance can be estimated through RLD in the 60–90 cm soil depth in dry-habitat genotypes such as DodR. Also, SH was identified as a good trait that could be used as a proxy to make estimations of RLD in bambara groundnut, and these could be prioritised for screening large populations for dry habitats. Overall, an increase in TRL allowed DodR to quickly explore water at a deeper soil depth in response to gradually declining soil water availability and high RLD in genotypes such as DodR, and S19-3 also offered adaptive advantage over other genotypes under DS. DodR and S19-3 can be used to map the genes and alleles responsible for root trait regulation and potential root system plasticity, shedding light on their evolution and ecological significance.

## Data Availability Statement

The datasets presented in this study can be found in online repositories. The names of the repository/repositories and accession number(s) can be found in the article/Supplementary Material.

## Author Contributions

KIM, HHC, SM, and FM conceived and designed the experiments. KIM performed the experiments, analysed the data, and wrote the main body of the manuscript. HHC, SM, and FM revised the manuscript. All authors contributed to the article and approved the submitted version.

## Conflict of Interest

The authors declare that the research was conducted in the absence of any commercial or financial relationships that could be construed as a potential conflict of interest.

## Publisher’s Note

All claims expressed in this article are solely those of the authors and do not necessarily represent those of their affiliated organizations, or those of the publisher, the editors and the reviewers. Any product that may be evaluated in this article, or claim that may be made by its manufacturer, is not guaranteed or endorsed by the publisher.
