# Supplementary material for: Natural Genotypic Variation Underpins Root System Response to Drought Stress in Bambara Groundnut [Vigna subterranea (L.) Verdc.]
Source: Front Plant Sci. 2022 Mar 28;13:760879. doi: 10.3389/fpls.2022.760879 (PMC8996172; doi:10.3389/fpls.2022.760879)
Supplement: Supplementary file 1 [file Data_Sheet_1.docx]

Supplementary Material

## Supplementary Figures

**
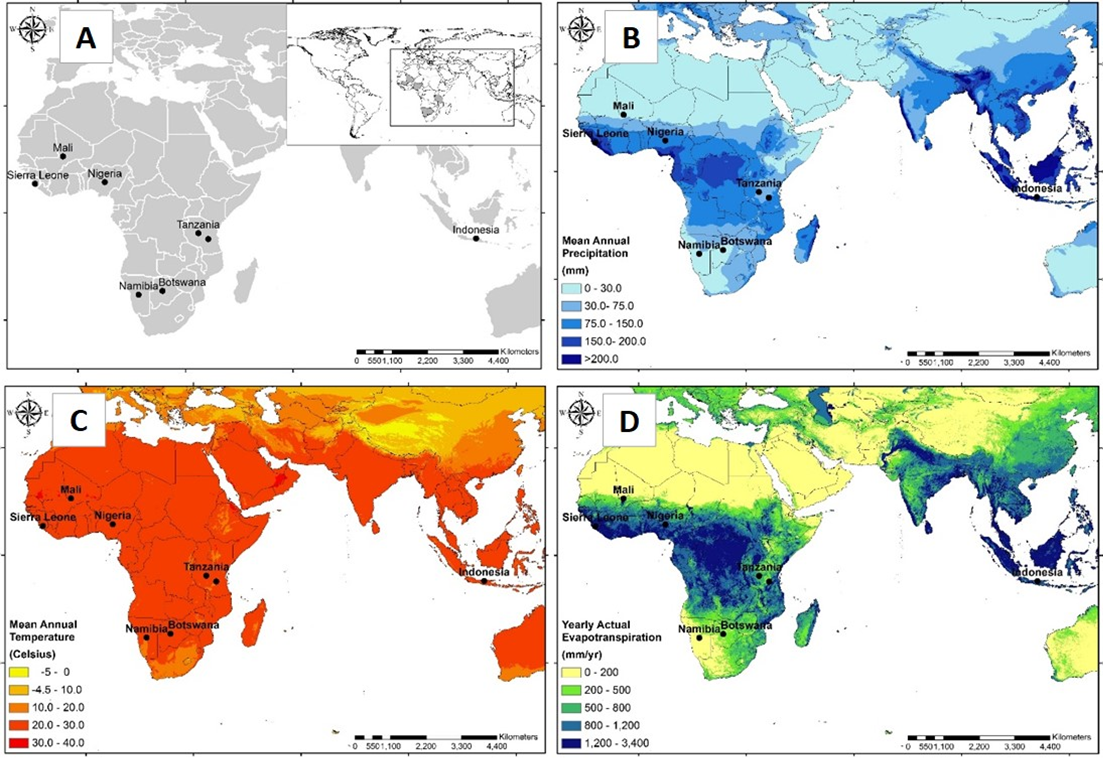
**

**Supplementary Figure 1.** Location of origin for the eight bambara groundnut (Vigna subterranea (L.) Verdc.) genotypes [west Africa (n = 3), east Africa (n = 2), southern Africa (n = 2), and southeast Asia (n = 1)]. (A) Close-up of Africa and southeast Asia collection points (B) Mean annual precipitation (C) Mean annual temperature and (D) Mean annual evapotranspiration. Plotted with the package raster (Fick and Hijmans 2017).


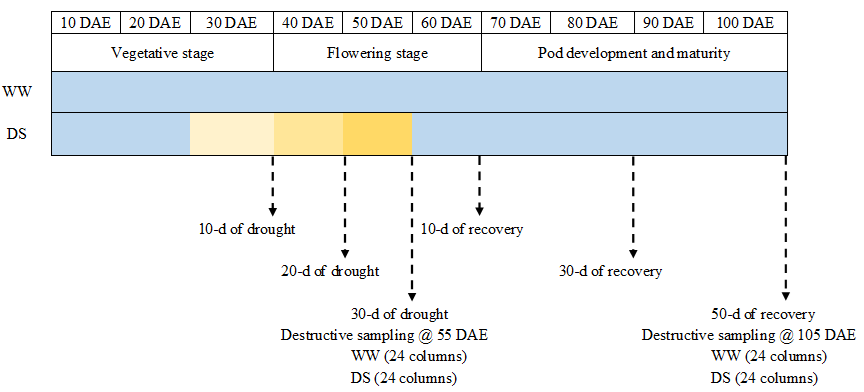


**Supplementary Figure 2.** Design of the study: drought stress (DS) treatments were applied before and during the flowering stage by withholding irrigation. The DS treatment was maintained for 30-d followed by re-watering. Well-watered (control) was designated as WW treatment and received irrigation (to field capacity) throughout the growth period. The period of WW and DS treatment is represented by the solid ungraded blue colour and a graded brown colour scheme indicating an increasing DS intensity, respectively.


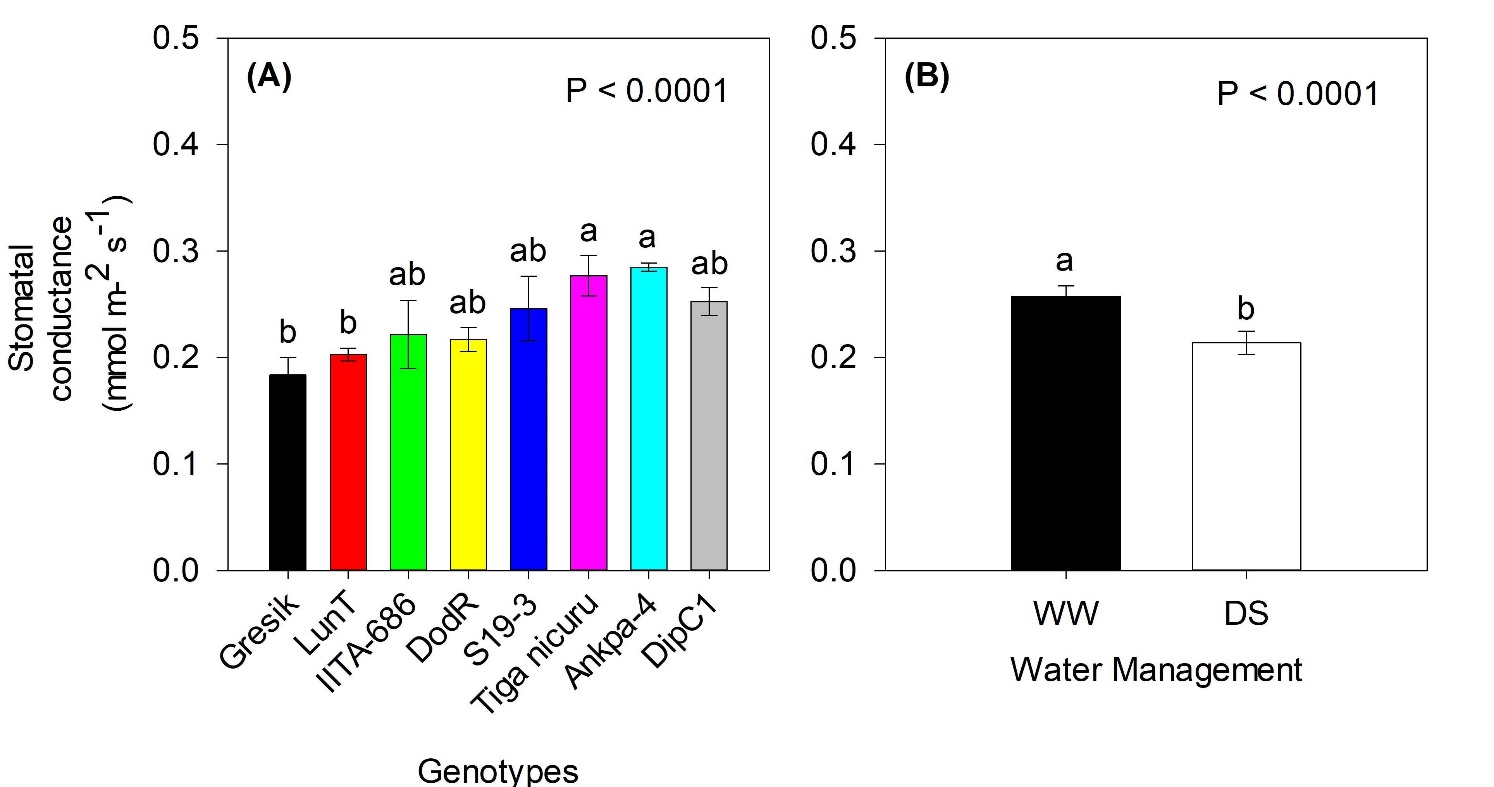


**Supplementary Figure 3**. Effect of Genotype (G) — (A), the data is mean ± se values (n = 6) and Water management (WM) — (B), the data is mean ± se values (n = 24) at 35 DAE on stomatal conductance, gs (mmol m^−2^ s^−1^) of eight bambara groundnut genotypes during the 2018 season. The data is mean ± se values (n = 6) with different letters showing significant differences by Tukey's honest significant difference post hoc test for treatments.


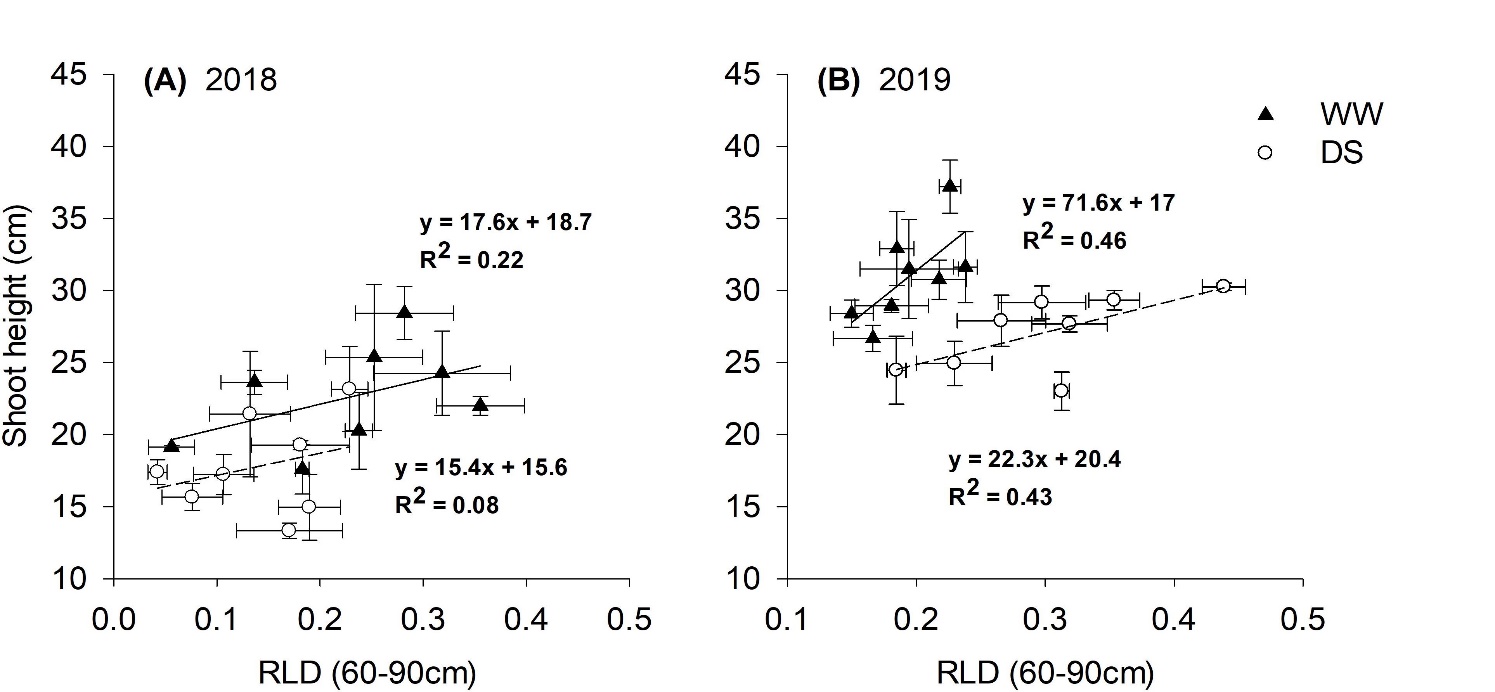


**Supplementary Figure 4**. Relationship between root length density (RLD 0-30cm) and shoot height (cm) for two seasons: 2018 (A) and 2019 (B) under well-watered conditions (WW) and drought stress (DS). Coefficient of determination R2 reported upon fitting with equation y = a*x + y0.

**Supplementary Figure 5**. Relationship between root length density (RLD 0-30cm) and grain yield (g plant^-1^) for two seasons: 2018 (A) and 2019 (B) under well-watered conditions (WW) and drought stress (DS). Coefficient of determination R2 reported upon fitting with equation y = a*x + y0.
